# STRATEGIES USED TO HELP OLDER ADULTS ACCESS VIDEO VISITS DURING COVID-19: A SYSTEMATIC REVIEW

**DOI:** 10.1093/geroni/igad104.2313

**Published:** 2023-12-21

**Authors:** Landon Haller, Rena Rodriguez, Lindsay (Na Yeon) Park, Kenneth Lam

**Affiliations:** UCSF, San Francisco, California, United States; University of California, San Francisco, San Francisco, California, United States; University of California, San Francisco, San Francisco, California, United States; Division of Geriatrics, San Francisco, California, United States

## Abstract

Many articles have described how older adults struggled to access telemedicine during the COVID-19 pandemic, but few have described how that digital divide was bridged. To comprehensively describe strategies used to help older adults access telemedicine during the pandemic, we conducted a systematic review of 4 databases (PubMed, EMBASE, CINAHL, and PsycInfo) for English articles (search terms: “tele*”, “mHealth”, “eHealth”, “video*”, “65 and older”) published from March 2020 to July 2022. We included articles that described any effort to help older persons (or a predominantly older population) access synchronous video visits during the pandemic, without requirements for study design. We categorized strategies as micro-, meso-, or macro-level per the Ecological Systems Theory framework to differentiate strategies implemented by clinicians, health systems, and policymakers. From 3799 records, we included 134 articles published in 2020 (23%), 2021 (51%), and 2022 (26%); most (57%) described strategies from the US. The most common specialties were Geriatrics (24%), Other (i.e. Occupational Therapy; 18%), and Medical Subspecialties (i.e. Cardiology, Oncology; 11%). Macro-level interventions included waivers for privacy regulations and increased reimbursement for telemedicine. Meso-level interventions included mobile vans offering devices and internet connection and protocols to test visits in advance. Micro-level interventions included written signs to troubleshoot sound and adapting clinical examinations virtually. We found many interventions at different levels of society to help older adults access video visits during the pandemic. Appraisals of the feasibility of telemedicine in older persons should consider the extent to which these strategies are used.

